# Effectiveness of advanced nursing care (ANC) on bone cancer pain, psychological disorders and quality of life in patients with primary bone cancers: A protocol for a PRISMA-compliant meta-analysis

**DOI:** 10.1097/MD.0000000000022711

**Published:** 2020-10-23

**Authors:** Lekun Li, Yujie Liu, Xiaofeng Ren, Kai Qu, Xiaona Liu

**Affiliations:** a Department of Spinal Surgery; b Department of Nursing, Liaocheng People's Hospital, Liaocheng, Shandong Province; c Department of Hepatobiliary Surgery, The First Affiliated Hospital of Xi’an Jiaotong University, Xi’an, Shaanxi Province, China.

**Keywords:** advanced nursing care, bone cancer pain, primary bone cancers, psychological disorder, quality of life

## Abstract

**Background:**

Advanced nursing care (ANC) has been reported to effectively relieve bone cancer pain, prevent psychological disorders and improve the quality of life (QoL) in patients with primary bone cancers (PBC) during the treatment. However, the exact effect of ANC remains controversial. This systematic review will aimed to assess the effectiveness of ANC on bone cancer pain, psychological disorders and QoL in patients with PBC.

**Methods:**

Eligible randomized controlled trials (RCTs) and high-quality prospective cohort studies were searched from Excerpt Medica Database (Embase), PubMed, Google Scholar, Medline, Cochrane Library, Web of Science (WOS), China National Knowledge Infrastructure (CNKI), Chinese Bio Medical Database (CBM), China Scientific Journal Database (CSJD), and Wanfang Database. Papers in English or Chinese published from January 2000 to July 2020 will be included without any restrictions. The clinical outcomes including bone cancer pain, psychological disorders, QoL, and adverse events of ANC in patients with PBC were systematically evaluated.

Two reviewers will separately carry out study selection and data extraction. Stata 14.0 and Review Manager 5.3 were used for data analysis. Methodological quality for each eligible clinical trial will be assessed by using Cochrane risk of bias tool. Subgroup and meta-regression analysis will be carried out depending on the availability of sufficient data.

**Results:**

This study will comprehensively summarize all potential evidence to systematically investigate the effects and safety of ANC on bone cancer pain, psychological disorders and QoL in patients with PBC.

**Conclusion:**

The findings of this study will help to determine whether ANC is effective or not on bone cancer pain, psychological disorders and QoL in patients with PBC.

**INPLASY registration number:**

INPLASY202090037.

## 1 Introduction

### 1.1 Description of the background

Primary bone cancers (PBC) include osteosarcoma (OS), Ewing sarcoma (ES), and chondrosarcoma (CS), accounting for less than 0.2% of all cancers.^[1–3]^ Timely diagnosis is challenging because of nonspecific symptoms that mimic common musculoskeletal injuries, and low suspicion by physicians.^[1]^ OS is one of the most frequent primary sarcoma of bone among young population.^[4–7]^ It typically develops in the metaphysis of long bones, specifically the distal femur, proximal tibia, and proximal humerus.^[1,4–7]^ Current treatment strategy usually consists of several weeks of chemotherapy before the surgery, then following by the surgery, and also several weeks of chemotherapy after the surgery.^[4,6,7]^ However, the overall outcome results were disappointed and unsatisfied during the past decades.^[4,6,7]^ ES is the second most common PBC and is similar to OS in terms of presenting symptoms, age at occurrence, and treatment.^[1]^ Late complications and secondary malignancies is the main problem for ES treatment.^[8,9]^ After treatment, patients with ES require very long-term follow-up in order to detect secondary malignancies and growth-related musculoskeletal complications.^[8,9]^ CS is the rarest PBC, primarily affecting adults older than 40 years.^[10–14]^ It constitutes a heterogeneous group of PBC characterized by hyaline cartilaginous neoplastic tissue.^[11–14]^ Survival rates are higher because most of these tumors are low-grade lesions.^[11–14]^ Pain is the first clinical symptom of cancer in a large population of cancer patients, particularly in advanced cancer patients, which strongly affect all aspects of patients’ life (such as mood, sleep, relationships, and walking ability).^[15–18]^ Tumor-derived, inflammatory, and neuropathic factors may simultaneously contribute to cancer pain, such as bone cancer pain.^[17,19]^ In addition, most cancer patients who received traditional chemoradiotherapy also experience more psychological disorders, such as depression and anxiety.^[20–22]^ The unpleasant side effects of PBC treatment are also affect the quality of life (QoL) of patients.

### 1.2 Description of the intervention

**Figure 1:**
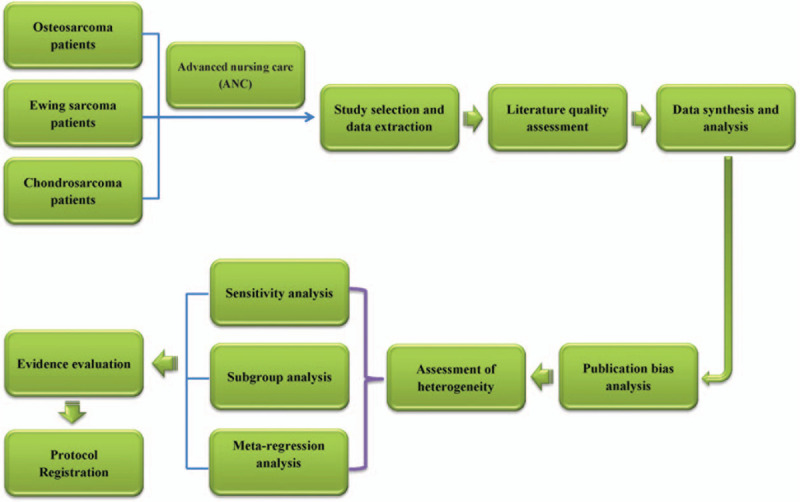
Work flow of the present study.

Currently, advanced nursing care (ANC) plays an increasingly important role in the comprehensive treatment of PBC.^[23–27]^ Accumulating evidence suggests a nurse-led disease management program may provide more comprehensive care, including symptom management, psychological and/or social support, lifestyle changes, and health education et al.^[28–30]^ It has been reported to effectively relieve the pain caused by cancer, prevent psychological disorders and improve the QoL in patients with PBC in several studies.^[23,24,29,31]^ Unfortunately, no study has systematically assessed these effects of ANC for PBC. Therefore, in this study, we will systematically evaluated the effectiveness of ANC on bone cancer pain, psychological disorders, and QoL in patient with PBC including OS, ES, and CS through the meta-analysis, in order to provide scientific reference for the design of future clinical trials (Work flow of the present study, Fig. 1).

- **Review question:** Whether ANC is effective or not on bone cancer pain, psychological disorders and QoL in patients with PBC.
- **Study aim/Objective:** The aim of our study is to systematically investigate the effectiveness of ANC on bone cancer pain, psychological disorders and QoL in patients with PBC.

## 2 Methods

This study has been registered on the International Platform of Registered Systematic Review and Meta-Analysis Protocols (INPLASY), and will be conducted according to the Preferred Reporting Items for Systematic Reviews and Meta-Analyses Protocols (PRISMA-P) guidelines.^[32]^ The registration number was INPLASY202090037 (DOI number is 10.37766/inplasy2020.9.0037, https://inplasy.com/inplasy-2020-9-0037/). No ethic approval is required for this study, because all the data will be extracted from previous published studies.

### 2.1 Search strategy

To perform a comprehensive and focused search, experienced systematic review researchers will be invited to develop a search strategy. The plan searched terms are as follows: “bone cancer” or “primary bone cancers” or “cancer in the bones” or “osteosarcoma” or “Ewing sarcoma” or “chondrosarcoma” or “BC” or “OS” or “ES” or “CS” and “pain” or “cancer pain” or “bone cancer pain” or “quality of life” or “QoL” or “psychological disorder” or “adverse events” and “nursing care” or “advanced nursing care” et al. The detailed sample of search strategy for PubMed database is shown in Table 1. Similar search strategies will be modified and used for the other databases.

**Table 1:**
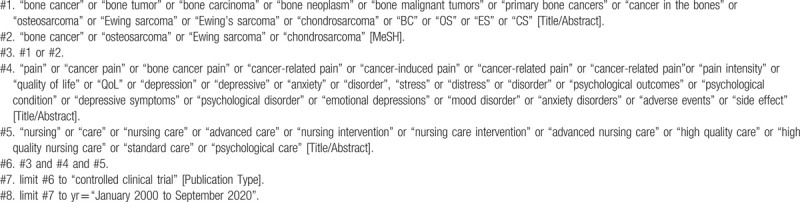
Searching strategy in PubMed.

### 2.2 Eligibility criteria

#### 2.2.1 Types of studies

All available randomized controlled trials (RCTs) and high-quality prospective cohort studies that assessed the effectiveness of ANC on bone cancer pain, psychological disorders and QoL in patients with PBC will be included in this systematic review.

#### 2.2.2 Types of participants

OS, ES, and CS patients who had severe bone cancer pain, or clinically diagnosed depression disorder or poor QoL will be included in this study, without restrictions of country, race, gender, etc.

#### 2.2.3 Types of interventions

In the experimental group, all patients must receive ANC for bone cancer pain, psychological disorders or for improving the QoL.

#### 2.2.4 Comparator

The control intervention can be any therapies, except ANC.

#### 2.2.5 Exclusion criteria

Articles without sufficient available data, non-comparative studies, case reports, and series, literature reviews, meta-analysis, letter to the editor, and other unrelated studies will be all excluded from analysis.

### 2.3 Information sources

Electronic databases including Excerpt Medica Database (Embase), PubMed, Google Scholar, Medline, Cochrane Library, Web of Science (WOS), China National Knowledge Infrastructure (CNKI), Chinese Bio Medical Database (CBM), China Scientific Journal Database (CSJD), and Wanfang Database, will be systematically searched for eligible studies from January 2000 to September 2020. In addition, we will also identify conference proceedings, reference lists of included studies, and websites of clinical trials registry. Language is limited with English and Chinese.

### 2.4 Types of outcomes

#### 2.4.1 Main outcomes

The primary outcomes will include total pain relief rate and QoL.

- Total pain relief rate. The reduction in pain intensity was measured using a visual analogue scale (VAS), verbal rating scale, or numerical rating scale (NRS). The intensity of pain was evaluated by the World Health Organization (WHO) standards with NRS, and expressed as numerical numbers ranging from 0 (for no pain) to 10 (for extreme pain).
- QoL which is assessed using Karnofsky performance score (KPS) or any other associated scales or scores.

#### 2.4.2 Additional outcomes

The secondary outcomes comprise of psychological outcomes and adverse events.

- Psychological outcomes. Depression will be measured by using the Hamilton Depression Rating Scale or any relevant scales; Anxiety will be measured by using the Hamilton Anxiety Rating Scale or other tools.
- Adverse events. Any expected or unexpected adverse events are measured according to WHO standards.

### 2.5 Study selection and data extraction

We will pool the evidence according to Cochrane Handbook for Systematic Reviews of Interventions to.^[33]^

#### 2.5.1 Study selection

**Figure 2:**
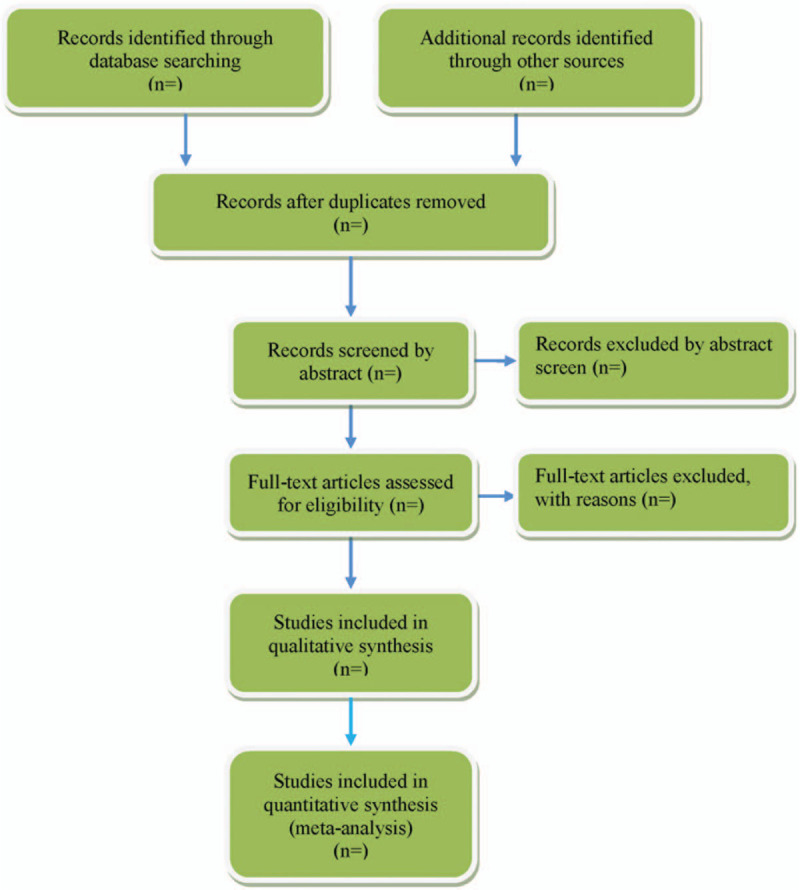
Study selection process for the meta-analysis.

Endnote X7 software will be used for literature managing and records searching. Two experienced authors (Lekun Li and Yujie Liu) will be reviewed independently to identify potential trials by assessing the titles and abstracts. The full text will be further reviewed to determine potential eligible studies. Disagreements between the 2 authors will be resolved by discussing with the third investigator (Xiaofeng Ren). A PRISMA-compliant flow chart (Fig. 2) will be used to describe the selection process of eligible literatures.

#### 2.5.2 Data extraction

Two investigators (Lekun Li and Yujie Liu) will be responsible for the data extraction independently. The following data will be extracted from eligible literatures:

- Study characteristics: first authors name, year of publication, country of study, sample size, study methods (such as randomization, blinding, etc.) and follow-up duration, et al.
- Participant characteristics: age, gender, ethnicity, KPS score, pain score, severity of psychological disorder, inclusion, and exclusion criteria, et al.
- Interventions: intervention methods and duration of intervention, et al.

- **Dealing with missing data:** When any data are missing or insufficient, we will contact original authors by using email. If those relevant data are not acquired, we will only analyze the available data, and discuss its impact as a limitation.

### 2.6 Risk of bias assessment

Two experienced authors (Lekun Li and Yujie Liu) will assess the risk of bias for each eligible trial according to the guidance of the Cochrane Handbook for Systematic Review of Interventions independently.^[33,34]^ This tool comprises of 7 items including selection, selection, performance, detection, attrition, reporting and other bias, and each item is further divided as 3 different levels: high, unclear, or low risk of bias. EPOC guidelines will be used to assess the risks of non-RCTs.^[35]^ Any disagreements will be resolved via discussion with a third researcher (Xiaofeng Ren).

### 2.7 Data synthesis and analysis

Stata 14.0 (Stata Corp, College Station, TX, USA) and Review Manager 5.3 (Nordic Cochran Centre, Copenhagen, Denmark) statistical software were used for statistical analyses. Continuous data will be presented as standardized mean difference (SMD) with their 95% confidence intervals (CIs), and dichotomous data will be recorded as risk ratio (RR) with 95% their CIs. A two-tailed *P* < .05 was considered statistically significant.

### 2.8 Assessment of heterogeneity

Cochrans Q and Higgins *I*^*2*^ statistic were used to assess heterogeneity among the included clinical trials. *P* < .1 for the Chi^2^ statistic or an *I*^*2*^ > 50% will be considered as showing considerable heterogeneity.^[36]^ A fixed effect model will be used to calculate the outcomes when statistical heterogeneity is absent; otherwise, the random effects model will be used for analysis.

### 2.9 Subgroup and meta-regression analysis

If the data are available and sufficient, subgroup and meta-regression analysis will be conducted to explore the source of heterogeneity with respect to location, study quality, intervention types, and treatment duration.

### 2.10 Sensitivity analysis

Sensitivity analysis will be carried out to assess the reliability and robustness of the aggregation results by eliminating low quality or high bias risk trials. A summary table will report the results of the sensitivity analyses.

### 2.11 Publication bias analysis

If the included studies are sufficient (≥10 trials), we will detect publication biases of included trials using funnel plots, Beggs and Egger regression test.^[37–39]^ If publication bias existed, a trim-and-fill method should be used to coordinate the estimates from unpublished studies, and the adjusted results were compared with the original pooled RR.^[40]^

### 2.12 Evidence evaluation

The quality of evidence and the strength of the main result recommendations will be determined by using the guidelines of the Grading of Recommendations, Assessment, Development, and Evaluation (GRADE). The quality of all evidence will be evaluated as high, moderate, low, and very low levels respectively.^[41]^

### 2.13 Dissemination plans

We will disseminate the results of this systematic review by publishing the manuscript in a peer-reviewed journal.

## 3 Discussion

Over the past 30 years, treatment advances and the addition of neoadjuvant chemotherapy have led to improved 5-year survival in patients with PBC.^[42]^ Unfortunately, most patients with PBC will suffer from severe bone cancer pain and depression disorder, which seriously affects the QoL. Although several managements can help relieve bone cancer pain, psychological disorder and improve QoL in PBC patients, but it is not always effective for some patients.^[23,24,29,31,43]^ Therefore, therapies that could significantly relieve cancer-related pain, improve psychological health condition and QoL are what we need to pursue now.

### 3.1 Strengths and limitations of this study

Even though there was statistical analysis of published clinical trials, the exact therapeutic effects of ANC on bone cancer pain, psychological disorders and QoL in patients with PBC were remains controversial. This systematic review will provide a helpful evidence for clinicians to formulate the best nursing strategies for PBC patients with bone cancer pain, psychological disorder and poor QoL, and also provide scientific clues for researchers in this field. There may be a language bias with the limitation of English and Chinese studies. Individual differences in patients and diverse intervention types among included trials may also cause a certain degree of heterogeneity.

## Author contributions

**Conceptualization:** Lekun Li, Xiaona Liu.

**Data curation:** Lekun Li, Yujie Liu, Xiaofeng Ren.

**Formal analysis:** Lekun Li, Yujie Liu, Xiaofeng Ren.

**Funding acquisition:** Kai Qu.

**Investigation:** Lekun Li, Yujie Liu, Xiaofeng Ren.

**Methodology:** Lekun Li, Yujie Liu, Xiaofeng Ren, Kai Qu.

**Project administration:** Lekun Li, Xiaona Liu.

**Resources:** Lekun Li, Xiaona Liu.

**Software:** Lekun Li, Xiaona Liu.

**Supervision:** Lekun Li, Xiaona Liu.

**Validation:** Lekun Li, Kai Qu, Xiaona Liu.

**Visualization:** Lekun Li, Yujie Liu, Xiaofeng Ren.

**Writing – original draft:** Lekun Li, Yujie Liu, Xiaofeng Ren.

**Writing – review & editing:** Lekun Li, Kai Qu, Xiaona Liu.
